# Perceived research burden of a novel therapeutic intervention: A study of transcranial magnetic stimulation for smoking cessation

**DOI:** 10.3389/fresc.2023.1054456

**Published:** 2023-03-03

**Authors:** Alina Shevorykin, Ellen Carl, Amylynn Liskiewicz, Colleen A. Hanlon, Warren K. Bickel, Martin C. Mahoney, Darian Vantucci, Lindsey Bensch, Hannah Thorner, Matthew Marion, Christine E. Sheffer

**Affiliations:** ^1^ Department of Health Behavior, Roswell Park Comprehensive Cancer Center, Buffalo, NY, United States; ^2^ Department of Cancer Biology, Wake Forest School of Medicine, Winston-Salem, NC, United States; ^3^ Addiction Recovery Research Center, Center for Health Behavior Research, Fralin Biomedical Research Institute at VTC, Roanoke, VA, United States

**Keywords:** participant research burden, transcranial magnetic stimulation, smoking cessation, treatment, rTMS

## Abstract

**Background:**

Translating repetitive transcranial magnetic stimulation (rTMS) into evidence-based clinical applications relies on research volunteers with different perspectives on the burden of study participation. Additionally, clinical applications of rTMS require multiple visits over weeks or months, the impact of research burden is an important component for these studies and translation of these findings to clinical practice. High frequency rTMS has significant potential to be developed as an evidence-based treatment for smoking cessation, however, the optimal rTMS dosing strategies have yet to be determined. Participant burden is an important component of determining optimal dosing strategy for rTMS as a treatment for long-term smoking cessation.

**Methods:**

In this double-blinded, sham-controlled, randomized design, the effects of treatment duration, intensity, and active/sham assignment of rTMS on research burden were examined.

**Results:**

Overall level of perceived research burden was low. Experienced burden (*M* = 26.50) was significantly lower than anticipated burden (*M* = 34.12). Research burden did not vary by race or income.

**Conclusions:**

Overall research burden was relatively low. Contrary to our hypotheses, we found little evidence of added significant burden for increasing the duration or intensity of rTMS, and we found little evidence for differences in research burden by race or income.

**Clinical Trial Registration:**

identifier NCT03865472.

## 1. Introduction

“None knows the weight of another’s burden.” — George Herbert

Tobacco use continues to be one of the greatest preventable causes of death and disease in the world today (1, 2). In the US, smoking cigarettes causes nearly half a million deaths annually, including nearly one third of all cancer deaths (1, 3). Most smokers make repeated unsuccessful attempts to quit every year (4), and while evidence based medications and behavioral interventions are available, considerable opportunities to improve treatment efficacy remain (5). Moreover, cigarette smoking is increasingly concentrated among many marginalized groups, including racial minorities and those with limited financial resources, who are typically underrepresented in smoking cessation research (1, 6). Clinical research is needed to develop new and/or to enhance existing therapies to advance the treatment of cigarette smoking and other tobacco product use, especially among those groups where disparities in tobacco use persist. Repetitive Transcranial Magnetic Stimulation (rTMS) was recently cleared by the FDA for short-term smoking cessation (FDA K200957), however more research is needed to determine the optimal dosing of rTMS to achieve long-term abstinence. Given that multiple rTMS sessions per week over the course of several weeks are needed for efficacy, and long-term smoking cessation outcomes require at least 6 months of longitudinal observation, evaluating participants' perception of the burden associated with this approach is essential to the continued investigation of rTMS for long-term smoking cessation.

All clinical research relies on individuals' voluntary participation. These individuals come from different backgrounds, have different motivations for participation, and are likely to have different perspectives on the burdens and benefits of participation (7). Participant research burden is defined as “a subjective phenomenon that describes the perception by the participant of the psychological, physical, and/or economic hardships associated with participation in the research process” (8). Contemporary conceptualizations of research burden focus on the subjective, perceived nature of the research burden, similar to perceived nature of stress. For instance, experiences can be highly stressful for one person and not at all stressful for another. Similarly, the same number of rTMS sessions might not be perceived as similarly burdensome to different participants. Nonetheless, research burden has the power to significantly impact the research process when participants perceive that their resources to be inadequate or insufficient to handle the demands of study participation (9). Research burden might also be mediated by the relevance of the research topic to individuals' health or motivations, the health status of participants, and the characteristics of the research environment (10). Understanding research burden is a methodological necessity in the rigorous development of rTMS for long-term smoking cessation because important factors such as dosing must be understood in the context of participants' willingness to complete the treatment and the long-term outcome assessments.

Research burden also has implications that affect biases in recruitment, retention, and ultimately the generalizability of findings. Recruitment challenges can delay the study process, cause budgetary challenges, and prevent study completion. In smoking cessation trials, difficulties with retention result in missing data and a reduction in the accuracy of efficacy estimates (11, 12) and may lead to non-significant findings in an intent-to-treat design. Participant fatigue from onerous baseline or outcome assessments can affect the accuracy of assessments (13). Disproportionate challenges with research burden for particular groups can affect sample characteristics and generalizability of findings (14). Results need to be generalizable to a significant proportion of individuals who smoke cigarettes today who often have physical and/or mental health conditions, have fewer financial or other resources, and/or belong to marginalized groups that harbor rightful distrust of the clinical research community (2, 3, 15). Understanding the experience of research burden among different participant groups is key to robust and generalizable findings.

We examined perceived research burden throughout the research process in an ongoing study investigating 20 Hz rTMS dosing strategies for long-term smoking cessation (16, 17). The parent study utilized a fully crossed, 3 × 2 × 2 randomized double-blinded, sham-controlled factorial design to examine the optimal dose of rTMS in terms of treatment duration and intensity in preparation for a large efficacy study. Many aspects of this study have the potential to negatively impact research burden including the number of in-person assessment appointments (*n* = 7), the number of rTMS sessions (8, 12 or 16 days of sessions), and the number of sessions per day (one or two sessions per day), as well as the possibility of being assigned to sham rTMS.

The COVID-19 pandemic had a significant impact on participants' ability to participate in the parent study. In addition, once the study resumed with new COVID-19 safety protocols, additional factors were introduced that could potentially affect research burden such as more complex travel, building access requirements, concerns about social distancing, and bothersome safety measures and protocols. To enable us to determine the degree of participant burden in rTMS study without extra stressors of the pandemic, we sought to examine research burden among participants who completed the research process before the onset of the pandemic. Among this group of participants, we compared anticipated research burden (ARB), assessed at baseline, with experienced research burden (ERB), collected at the final 6-month outcome assessment, to evaluate potential differences between anticipated and experienced burden. We hypothesized that increases in the number of days of treatment (i.e., duration) and the number of sessions per day (i.e., intensity) would be associated with increased perceived burden of research. We also hypothesized that racial minorities and lower income participants would experience significantly more research burden than white higher-income participants. These findings provide preliminary evidence for perceived research burden among participants enrolled in a rTMS study for long-term smoking cessation, a pre-pandemic comparison for any data collected during or after the pandemic. To our knowledge, this is the first documented evidence of perceived research burden from participants enrolled in any rTMS study.

## 2. Methods

### 2.1. Participants

Participants were right-handed adults (age 18–65) who smoked between 5 and 25 cigarettes per day and were motivated to quit smoking, tested negative on a 12-panel urine test for drugs of abuse and a pregnancy test (women only), and passed the Transcranial Magnetic Stimulation Adult Safety and Screening Questionnaire (18). Only participants who completed the study timeline before March 20, 2020, the date New York State was declared the epicenter of the COVID pandemic in the United States, were included. This study was approved by the Roswell Park Comprehensive Cancer Center Institutional Review Board in Buffalo, NY. All participants provided informed consent. Participants were compensated $20 for each in-person visit (i.e., baseline, MRI, rTMS sessions, outcome assessments). Participants were compensated an additional $50 bonus for completing all assigned rTMS sessions in a given week and a $100 bonus for completing all five outcome assessments. Participants were recruited primarily through flyers in the community, social media, print advertisement, and word of mouth.

### 2.2. Design

This study employed a fully crossed, double-blinded, sham-controlled, 3 × 2 × 2 randomized factorial design. The three dosing factors examined were treatment duration (8, 12, or 16 days of stimulation), treatment intensity (900 or 1,800 pulses per day), and active/sham rTMS.

### 2.3. Procedure

After screening eligible participants completed the baseline assessment and scheduled for a structural MRI of the head. Before undergoing the MRI, we used the extended International 10–10 EEG electrode system to place a fiducial marker at the AF3 electrode position, the site of stimulation. If the MRI showed no abnormalities, participants were randomized and provided with 30 min of smoking cessation counseling over the telephone to support their quit attempt. On the day of the first stimulation session, participants were required to show biochemically validated 24 h of abstinence from smoking. Participants attended one or two rTMS sessions per day, four days per week. Those randomized to 8 days of stimulation completed their sessions within 2 weeks; 12 days of stimulation within 3 weeks, and 16 days of stimulation within 4 weeks. Each rTMS session delivered 900 pulses of 20 Hz rTMS. Actual stimulation time per session was approximately 16 min. During the first 8 days of stimulation, participants read the 8 Forever Free smoking cessation self-help booklets (19, 20). Uptake of the content of the booklets at home and during session was tracked throughout the study. Participants returned to the laboratory for 5 outcome assessments: 4, 8, 12, 18, and 24 weeks after the first stimulation session. Participants' compensation. Participants were compensated $20 for each study visit including baseline assessment, MRI appointment, rTMS sessions, and outcome assessments. Participants were provided a bonus of $50 if they attended all 4 scheduled rTMS sessions within a week. Participants were provided a bonus of $100 bonus if they completed all five outcome assessments.

### 2.4. Bioethics

The study was approved by the Institutional Review Board of Roswell Park Comprehensive Cancer Center (#I-65718). Informed consent was obtained from all participants.

### 2.5. Stimulation preparation

All participants were prepared identically. The first session of each week was initiated by determining the motor threshold (MT), a well-established method of standardizing stimulation. Stimulation was delivered at 110% of the MT. After determining MT, conductive skin preparation gel was placed on two rectangular, carbon-impregnated rubber electrodes (4 cm × 5 cm) placed firmly over the left frontalis muscle about 1 cm above the eyebrow underneath the headband that held the Brainsight (Rogue Research Inc) neuro-navigation reflective tracking balls. These electrodes delivered focal electrical stimulation for the active conditions but were not activated during the sham conditions.

### 2.6. Measures

Demographic characteristics, collected at baseline, included age, sex, race, ethnicity, partnered status, education, and household income. Research burden was assessed with the patient version of the Perceived Research Burden Assessment (PeRBA), a 21-item instrument designed to assess participant research burden in cancer clinical trials. A preliminary investigation of the PeRBA with decisionally intact patients visiting the University of Pittsburgh Alzheimer Disease Research Center using vignettes of research studies suggested high internal consistency (Cronbach's alpha .87–.96) and good convergent and discriminant validity (8). The PeRBA requires participants to rate burdensome statements on a 5-point Likert scale from strongly disagree (1) to strongly agree (5). The PeRBA has three scales: **Logistical** (e.g., “I felt that this study's visits might be too frequent”, “I feel that it might be inconvenient to get to the research center”), **Psychological** [e.g., “I feel that the researchers might ask me too many questions”, “I feel that I may become emotionally upset by the research procedure(s)”] and **Physical** (e.g., “I feel that I may be physically harmed by the research procedures or study intervention”, “I feel that I might experience side effects from the research procedures or study intervention”). The total score is a sum of all the PeRBA items, ranging from 21 to 105, with lower scores reflecting lower perceived burden. The range of the Logistical Burden scale was from 9 to 45; the Psychological Burden scale from 7 to 35; and Physical Burden scale from 5 to 25. In addition, we tracked number of rTMS sessions attended.

Other measures included nicotine dependence, stress, and depressive symptomology used to characterize the sample. Nicotine dependence was assessed with the 6-item Fagerström Test for Nicotine Dependence (FTND), range 0–10 (21). Stress level was assessed with the 4-item Perceived Stress Scale 4 (PSS-4) on a 5-point scale where 0 = “never” and 4 = “very often”, range 0–16 (22). Depressive symptomatology was assessed with the 20-item Centers for Epidemiological Studies Depression Scale (CES-D) on a 4-point scale where 0 = “rarely or none of the time” and 3 = “most or all of the time,” range 0–60. A score of 16 or greater indicates possible depression (23).

### 2.7. Statistical analyses

Descriptive analyses were used to characterize the sample. Cronbach's Alpha was used to examine the internal consistency of the PeRBA across all time points separately.

Repeated measures t-tests were used to examine differences between ARB, assessed at baseline, and ERB, assessed at the 6-month outcome assessment. A mixed-model repeated-measures analysis of variance (ANOVA) was used to examine the effects of duration (8, 12, or 16 days of TMS stimulation), intensity (900 or 1,800 pulses per day), and active/sham condition on PeRBA total scores. Race and household income were included in the model to examine whether participation disparately affected racial minorities and/or lower income participants. Race was dichotomized into White and non-white (e.g., Black, African American, Asian, Multi-racial, or Other). One-way between-subjects ANOVAs were conducted to compare the effects of race and household income on the proportion of missed rTMS sessions and missed outcome assessments. To guard against violations of the sphericity assumption with repeated-measures data, all main effects and interactions were reported as significant after the Greenhouse-Geisser correction. Means with standard errors, *F* tests and *p* values are reported (*α* < 0.05). All analyses were conducted in IBM SPSS, Version 23 (24).

## 3. Results

Participants (*n* = 51) in this study represented about 20% of the recruitment goal in the parent study. They were primarily middle-aged (*M* = 50.53, SD = 10.21). Two-thirds were female (62.7%) and two-thirds were White (68.6%). Nearly half of participants had household incomes less than $24,999 (48.0%). Most had some post-secondary education (64.7%), and about one-third worked full-time (35.3%). Participants smoked an average of 14.69 (SD = 5.44) cigarettes per day, were highly nicotine dependent (FTND, *M* = 5.06, SD = 1.89), reported moderate stress levels (PSS-4, *M* = 4.80, SD = 2.73) and no significant depressive symptomology (CES-D, *M* = 9.35 (SD = 6.53). See Table 1. Participants were nearly equally distributed between the active (45.1%, *n* = 23) and sham (54.9%, *n* = 28) conditions. See Table 2.

**Table 1:** Characteristics of participants (*n* = 51).

Characteristic	Category	Range or Levels	*M* (SD) or % (*N*)
Sociodemographic	Age (in years)	20–64	50.53 (10.21)
	Sex/Gender	Female	62.7 (32)
	Partnered status	Un-partnered	54.9 (28)
	Household income	≤$24,999	48.0 (24)
		$25,000–$49,999	21.6 (11)
		$50,000–$75,000	15.7 (8)
		>$75,000	15.7 (8)
	Education	≤High school	35.3 (18)
		College	56.9 (29)
		Graduate school	7.8 (4)
	Education (years)	10–19	13.91 (2.05)
	Hispanic	Yes	9.8 (5)
	Race	White	68.6 (35)
		Black or African American	19.6 (10)
		Asian	2.0 (1)
		Other or Multi racial	9.80 (5)
	Employment	Full-time	35.3 (18)
		Part-time	13.7 (7)
		Retired	15.7 (8)
		Disabled	9.8 (5)
		Unemployed	17.6 (9)
		Homemaker	7.8 (4)
Tobacco use At baseline	Cigarettes per day	6–30	14.69 (5.44)
	Age started smoking	8–44	16.94 (6.24)
	Years of regular smoking	4–50	30.73 (12.54)
	FTND	0–8	5.06 (1.89)
Psychosocial	PSS-4	0–11	4.80 (2.73)
	CES-D	0–28	9.35 (6.53)

Note: Unpartnered, single, divorced, separated, widowed; Partnered, married, partnered, or living with significant other; FTND, Fagerström Test for Nicotine Dependence; PSS-4, Perceived Stress Scale – 4 Item; CES-D, Center for Epidemiological Studies-Depression.

**Table 2:** Number of participants (*n* = 51) across duration and intensity.

Intensity	Duration		
	8 days	12 days	16 days
1 session per day	8 sessions (*n* = 11)	12 sessions (*n* = 10)	16 sessions (*n* = 9)
2 sessions per day	16 sessions (*n* = 9)	24 sessions (*n* = 6)	32 sessions (*n* = 6)

Intensity, 900 pulses provided during each session; Duration, number of days of stimulation.

**Figure 1:**
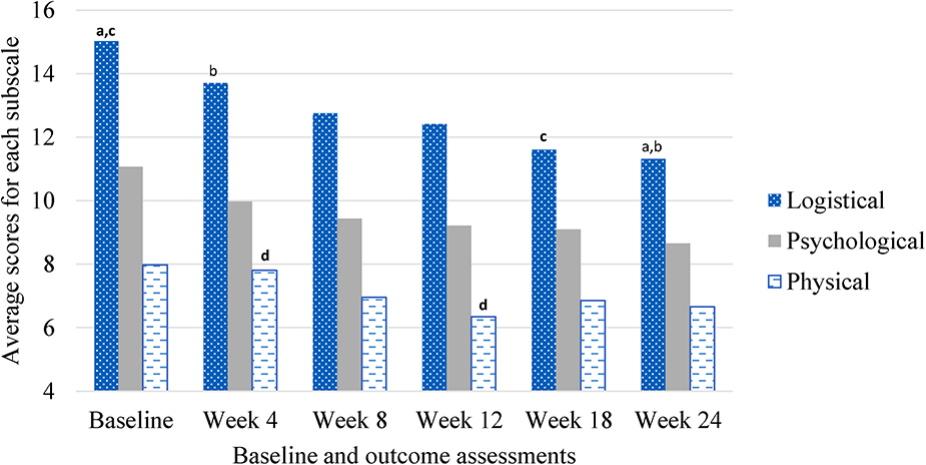
Perceived research burden subscales over time during rTMS treatment for smoking cessation. ^a^Same subscript reflects significant difference (*α* < .05).

The PeRBA showed similar internal consistency as reported in other studies (Cronbach's alpha overall = 0.978; Cronbach's alpha across assessment time points ranged from 0.931 to 0.965). The mean total burden was 31.68 (SD 7.86), Logistical Burden was 13.36 (SD 4.29), Psychological Burden was 9.76 (SD 2.72), and Physical Burden was 7.02 (SD 2.49). See Figure 1. The mean ARB (*M* = 34.12, SD = 15.62) was significantly greater than the mean ERB (*M* = 26.50, SD = 8.80, *t* = 3.53, *p* = .001).

Repeated-measures ANOVA revealed no main effects for time, (*M*_baseline _= 33.61, SE_baseline_ = 4.18, *M*_week 4 _= 31.41, SE_week 4_ = 1.47, *M*_week 8 _= 28.93, SE_week 8_ = 1.78, *M*_week 12 _= 27.83, SE_week 12_ = 1.50, *M*_week 18 _= 27.21, SE_week 18_ = 2.42, *M*_week 24 _= 26.22, SE_week 24_ = 2.11, *F* = 2.18, *p* = .15), active or sham condition, (*M*_active _= 28.74, SE_active_ = 2.69, *M*_sham_ = 29.57, SE_sham_ = 2.36, *F* = .19, *p* = .68), duration (*M*_8 days_ = 29.27, SE_8 days_ = 3.02, *M*_12 days_ = 29.71, SE_12 days_ = 2.95, *M*_16 days_ = 28.48, SE_16 days_ = 3.26, *F* = .06, *p* = .95), intensity (*M*_900pulses_ = 30.019, SE_900pulses_ = 2.29, *M*_1800pulses_ = 27.79, SE_1800pulses_ = 2.80, *F* = 1.27, *p* = .30), race (*M*_non−white_ = 29.18, SE_non−white_ = 2.90, *M*_white_ = 29.22, SE_white_ = 2.23, *F* = .07, *p* = .80), or income (*M*_under 15,000_ = 29.36, SE_under 15,000_ = 2.87, *M*_15,000–50,000_ = 28.14, SE_under 15,000–50,000_ = 3.26, *M*_above 50,000_ = 29.99, SE_above 50,000_ = 3.12, *F* = .17, *p* = .85) on perceived research burden. There were no interactions between time and active or sham condition (*F* = .16, *p* = .85), time and duration (*F* = .57, *p* = .69), time and intensity (*F* = 1.03, *p* = .38), time and race (*F* = .578, *p* = .57), time and income (*F* = .78, *p* = .55) on perceived research burden, see Table 3.

**Table 3:** The effects of duration, intensity, active/sham condition of rTMS, race and income on research burden across time.

Effect	Levels	Baseline	Week 4	Week 8	Week 12	Week 18	Week 24	*p*
Duration × Time	8 days	32.80	30.50	27.02	30.74	27.15	27.41	.687
	12 days	32.19	32.73	32.23	27.46	28.27	25.35	
	16 days	36.35	30.70	26.75	25.10	25.90	26.05	
Intensity × Time	900 pulses	36.34	32.00	30.33	27.08	28.46	26.95	.381
	1,800 pulses	29.71	30.57	26.93	28.89	25.43	25.18	
Condition × Time	Sham	31.92	31.24	31.03	28.08	29.34	25.79	.848
	Active	35.76	31.63	26.28	27.51	24.51	26.77	
Race × Time	White	32.74	31.05	29.18	26.76	28.51	27.08	.570
	Non-White	34.86	31.93	28.57	29.36	25.36	25.00	
Income × Time	Under $14,999	31.72	30.08	29.32	30.90	26.17	27.96	.553
	$15,000–$50,000	37.15	32.50	24.65	25.30	25.55	23.70	
	Above $50,000	32.64	32.00	32.36	26.50	29.96	26.46	

Overall, 85.9% of outcome assessments were completed with 86.7% completing the final 6-month outcome assessment. See Table 4. Race was not a significant predictor of missed rTMS sessions (*F* = 1.73, *p* = .15) or missed outcome assessments (*F* = .29, *p* = .91). Income was not a significant predictor of missed rTMS sessions (*F* = .58, *p* = .63) or missed outcome assessments (*F* = .06, *p* = .98).

**Table 4:** Percent of rTMS and outcome assessments completed by condition.

	Duration					
	8 days		12 days		16 days	
Intensity	900 pulses (1 session per day)	1,800 pulses (2 sessions per day)	900 pulses (1 session per day)	1,800 pulses (2 sessions per day)	900 pulses (1 session per day)	1,800 pulses (2 sessions per day)
Percent of rTMS sessions completed	100%	93.06%	100%	96.43%	91.67%	100%
Percent of outcome sessions completed	92.73%	68.9%	88%	100%	82.2%	86.7%

## 4. Discussion

Among participants who reached study end of a long-term rTMS smoking cessation trial prior to the COVID pandemic, overall research burden was relatively low. Contrary to our hypotheses, we found little evidence of added significant burden for increasing the duration or intensity of rTMS and we found little evidence for differences in research burden by race or income. The significant difference between ARB and ERB suggests that participants anticipated the burden of participation to be higher than they experienced by the end of the study. These preliminary findings suggest that increasing the number weeks of rTMS sessions from two to four and the number of rTMS sessions per day from one to two does not result in overburdening participants who are highly motivated to quit smoking, at least prior to the COVID-19 pandemic. The low number of missed sessions and the 87.7% retention rate provide convergent validity of this low level of research burden found among these participants. These findings also provide a pre-pandemic comparison for data collected during or post-pandemic.

Perceived participant burden is an important factor in the examination of optimal rTMS dosing for smoking cessation and other purposes. These findings suggest that the PeRBA might be a reasonable measure of participant burden in other multi-session rTMS studies. The internal consistency of the total scores in this study across time points ranged from 0.978 to 0.931, which compares favorably with the internal consistencies found in a preliminary study (0.87–0.96) as well as the level of burden anticipated from more invasive study procedures in other studies (7). Additional research into the psychometrics of the PeRBA might provide additional evidence of construct and criterion validity as well as reliability.

These findings are limited to participants who, of course, are willing to enroll in a lengthy research study to quit smoking. As usual, the screening and consent processes likely eliminated potential participants for whom the study appeared to be too burdensome. Research burden might also be mediated by the relevance of the research topic to individuals' health or motivations. Specifically, participants in this study were middle-aged individuals who were highly dependent on cigarette smoking, who expressed a strong desire to quit smoking, and who had experienced numerous failed quit attempts. These factors might also have affected perceived burden in this study in a positive manner.

The compensation participants receive for donating their time and personal data is an unexplored aspect of perceived research burden and may have contributed to any perceived inherent cost-benefit analyses that participants may have personally undergone. Timely and sufficient compensation might make study protocol compliance feel less burdensome. In this study participants were paid in cash immediately after meeting attendance requirements. In addition, the compensation schedule (i.e., including bonuses), which was designed to reinforce protocol compliance, might also have indirectly affected perceived burden in a positive manner. These factors might have added importance in this study because smoking in the United States and elsewhere is now highly concentrated among individuals with lower incomes. These individuals might not be able to attend if their time, travel, parking, and other expenses are not compensated. These factors suggest that attending to the amount, timeliness, and scheduling of compensation might make the study procedures feel less burdensome in addition to having other positive effects. More research is needed to quantify this important aspect of conducting research.

These findings might also reflect the characteristics of the research environment. A crucial element in managing perceived research burden is ensuring that individuals who express interest know what to expect throughout the research process. In addition, consistent and positive contact with research personnel, compelling financial compensation, effective tracking and reminder appointments, free parking, and flexible scheduling can contribute positively to perceived burden. Prior research links increased participant retention rates with research personnel who are organized, specialized, persistent and have good communication and interpersonal skills (25, 26). Adjusting to participant needs without altering study integrity is also likely to contribute to a reduced perception of burden. For this study, the research team thoroughly explained the research during the telephone screening conversation, during the in-person screening visit, as part of the consent process, and after the smoking cessation counseling session. Participants were provided with a written schedule, and were reminded by email, text, and telephone before all appointments. The team culture supported respect, professionalism, and compassion (e.g., addressing all participants as Mr. or Ms. unless invited otherwise; adopting a stance of unconditional positive regard; empathizing with challenges of quitting smoking). Additionally, the laboratory scheduled appointments from 7am until 7pm to accommodate participants' schedules and provide flexible scheduling if needed. Lower than anticipated levels of research burden may have been the result of any of these factors or their combination.

These preliminary findings have implications for the study of rTMS for smoking cessation and its translation to clinical practice. Furthermore, current findings have implications for our understanding of research burden, as well as the importance and relative ease of incorporating investigation of research burden into behavioral research in general, and as an important consideration in the examination and development of rTMS interventions in particular.

### 4.1. Strengths and limitations

Limitations in this study include a moderate sample size and the self-report nature of the PeRBA. However, the perceived nature of construct of participant burden recognizes that the same experience can be perceived differently by different individuals and is a strength as well. The assessment of perceived research burden did not include the role of participant compensation, the role of the reputation of the organization, or satisfaction with research staff and facilities. These factors should be considered in future studies.

## 5. Conclusions

Despite the high duration and intensity of rTMS exposure in the current study, we did not find evidence that these factors affect the perceived burden of rTMS in the research setting, or that the perceived burden of rTMS varies with race or income. Additionally, we found that experienced research burden, assessed during outcome assessment, was lower than the anticipated research burden evaluated at baseline, suggesting that appropriate preparation and accommodation on the part of the research team may have led to participant experiences of research burden that are in line with or lower that their anticipated burden. Findings suggest that there may be certain methodological considerations that can help improve perceived and/or actual research burden, which may in turn help improve overall study participation, retention, and other factors. Understanding research burden can help institutional review boards and other stakeholders to inform their decisions about implementation of novel approaches.

## Data Availability

The original contributions presented in the study are included in the article/Supplementary Material, further inquiries can be directed to the corresponding author.
